# Impact of the Diamond Light Source on research in Earth and environmental sciences: current work and future perspectives

**DOI:** 10.1098/rsta.2013.0151

**Published:** 2015-03-06

**Authors:** Ian T. Burke, J. Frederick W. Mosselmans, Samuel Shaw, Caroline L. Peacock, Liane G. Benning, Victoria S. Coker

**Affiliations:** 1 Earth Surface Science Institute, School of Earth and Environment, University of Leeds, Leeds LS2 9JT, UK; 2 Diamond Light Source Ltd, Harwell Science and Innovation Campus, Didcot, Oxfordshire OX11 0DE, UK; 3 School of Earth, Atmospheric and Environmental Sciences, University of Manchester, Manchester M13 9PL, UK

**Keywords:** environmental science, synchrotron, pollution, minerals, nanomaterials, chemical speciation

## Abstract

Diamond Light Source Ltd celebrated its 10th anniversary as a company in December 2012 and has now accepted user experiments for over 5 years. This paper describes the current facilities available at Diamond and future developments that enhance its capacities with respect to the Earth and environmental sciences. A review of relevant research conducted at Diamond thus far is provided. This highlights how synchrotron-based studies have brought about important advances in our understanding of the fundamental parameters controlling highly complex mineral–fluid–microbe interface reactions in the natural environment. This new knowledge not only enhances our understanding of global biogeochemical processes, but also provides the opportunity for interventions to be designed for environmental remediation and beneficial use.

## 1. Introduction

Ever since the first purpose-built synchrotrons were constructed in the early 1980s, a steadily increasing number of Earth and environmental scientists have used them to better understand the nature of the world around us and the consequences of anthropogenic activity. Reviews of some of the early work in the field have been made [1,2]. Initially, X-ray absorption spectroscopy (XAS) was a popular technique with its ability to determine the chemical environment of a particular element, e.g. Calas *et al*. [3] reviewed early studies of cationic environments in silicate glasses and minerals; in the late 1980s and early 1990s, studies using diffraction of mineralogical transformations at high pressure became widespread as the technology of high-pressure cells such as those using diamond anvils was developed [4]. The advent of third generation sources enabled a new raft of beamlines such as X-ray microprobes, which are used to map the speciation of trace elements in the environment [5].

Hence, there was much interest in the potential of Diamond among UK researchers. In 2001, a joint Franco-British meeting was organized at Molsheim to discuss which synchrotron beamlines would be most useful to molecular environmental science on the then proposed sources, Diamond and Soleil [6]. This led to a wish list of techniques and beamlines on the two respective synchrotrons. It was also recognized that many UK geoscientists were unaware of the possibilities that third generation sources offered, hence the Natural Environment Research Council and the Council for the Central Laboratories of the Research Councils sponsored the three-year Envirosynch project enabling UK based scientists to undertake experiments abroad to be able to better influence the choice and development of Diamond beamlines [7]. Many of the beamlines on the wish list have now been constructed or are in construction at Diamond (table 1).

**Table 1.** Diamond beamlines with extensive applications in the Earth and environmental sciences.

beamline	techniques	energy range (keV except where stated)	representative Earth and environmental science studies	year of first use
I15 extreme conditions	XRD under high pressure and temperature	20–80	high pressure study of barite [8]; study of the Fe–Si phase diagram [9]	2007
I18 microfocus spectroscopy	μXAS, μXRF and μXRD	2.1–20.8	elemental speciation in Ajka red mud [10]; Ni speciation in Cuban lateritic ores [11]	2007
I22 non-crystalline diffraction	SAXS and XRD	3.7–20	formation of green rust sulfate [12]; influence of Mg on monohydrocalcite crystallization [13]	2007
I11 high-resolution powder diffraction	powder XRD, long duration experiments	6–30	the aragonite–calcite transformation [14]; ikaite solubility in brines [15]	2008
I12 joint engineering, environmental and processing	X-ray tomography and high-energy diffraction imaging	50–150	*in situ* study of cracks in frozen soil [16]; thermal processing of Mg–Ca silicates [17]	2009
B22 multimode infrared imaging and microspectroscopy	IR microspectroscopy	0.0006–1.2 eV	functional group mapping of fungi on a mineral surface [18]; element variations in rhyolitic magma [19]	2009
B18 core EXAFS	XAS	2.05–35	adsorption of Cu to ferrihydrite [20]; the mobility of U in mining-impacted wetland [21]	2010
I13 imaging	phase contrast imaging and tomography	8–35		2012
I20 LOLA X-ray spectroscopy	XAS and X-ray emission spectroscopy	4–34		2012
I08 soft X-ray microscopy	SXM and XAS	250–4200 eV		2014
I14 hard X-ray nanoprobe	nanoXRF, nanoXRD and nanoXAS	4.5–25		2017

Two of the first seven Phase I beamlines at Diamond have proved popular in the environmental sciences: I18, the microfocus spectroscopy beamline, can be used to study heterogeneous materials with a micrometre-sized incident beam to collect absorption spectra from elements from P through to U and mapping the association of trace metals with host mineral phases using micro X-ray fluorescence (μXRF) [22]; and I15, the extreme conditions beamline, is designed for use as a high-pressure diffraction beamline operating using a 3.5 T wiggler source to provide X-rays with energies from 20 to 80 keV in either monochromatic or white beam form. It has the potential for laser heating experiments using diamond anvil cells.

Diamond Phase II built another 15 beamlines: including two more XAS beamlines: B18, the core XAS beamline, has an energy range from 2.05 to 35 keV [23], and I20, which has an energy range of 5–34 keV and is designed for the study of elements in trace concentrations and by emission spectroscopy, which gives more detail on the electronic structure of the absorbing atom [24]. A number of X-ray scattering and diffraction beamlines were added in Phase II. Those of particular relevance to Earth and environmental sciences include the small angle X-ray scattering (SAXS) beamline, I22, which is designed to look at distances between 0.1 and 500 nm, ideal for looking at crystallization from solution; the high-resolution X-ray diffraction (XRD) beamline, I11, which has an energy range up to 30 keV and can collect full XRD patterns in milliseconds with its suite of Mythen position-sensitive detectors [25]; the joint engineering, environmental and processing beamline, I15, which is used for high-energy X-ray tomography and diffraction imaging; and the long X-ray beamline, I13L, which has an imaging branchline, built in partnership with the University of Manchester, for direct and indirect transmission imaging on the micrometre scale [26]. The infrared (IR) microscope, B22, which is an extremely effective probe for revealing IR-active vibrational modes of molecular components at the microscopic scale, was also constructed in Phase II [27].

Diamond Phase III will build two beamlines for nano-imaging: the low-energy scanning transmission X-ray microscope, I08, with an energy range of 250–4000 keV that will be able to image on a 30 nm scale, and the hard energy nanoprobe, I14, which will image using the energy range 5–25 keV, and will be able to look at the environmental interactions of individual nanoparticles.

The ability to work with samples at near environmental conditions, at low elemental abundances and in complex matrices makes synchrotron-based analysis a particularly powerful tool for Earth and environmental scientists. The current capabilities of Diamond beamlines, illustrated in table 1, have already allowed a broad range of Earth and environmental processes to be studied, including: the environmental behaviour of contaminant metals and radionuclides, the processes controlling the cycling of elements in natural environments, mineral precipitation and weathering reactions, and the production of functional (nano)materials for environmental use. In addition, the important role of biological processes and interactions at microbial surfaces is a cross-cutting theme present in much of this research. The purpose of this paper is, therefore, to review current work in the Earth and environmental sciences that has used Diamond's beamlines during the first 5 years of operation and also to look to the future prospects for improved scientific understanding as new beamlines come online.

## 2. Contaminant processes and characterization of affected environments

Worldwide, mining and industrial processes have produced a complex legacy of contaminated land. The nature of the contaminants present and their environmental behaviour can be very specific to the process or materials involved. Traditional geochemical investigations (based on bulk chemical analysis) can describe the distribution of contaminants, but will not provide site managers and regulators with data that can be used to predict the likely environmental fate, bioavailability and risks caused by specific instances of contamination. Characterization of the exact chemical form(s) present and their physical associations in environmental materials goes a long way to providing a much more useful mechanistic understanding of contaminant behaviour. Often this involves using a combination of traditional chemical methods (e.g. sequential extractions or leaching tests (e.g. [28,29])), with spectroscopic investigations and high-resolution microscopy (e.g. [30–34]). Using synchrotron-based techniques has a distinct advantage over many other methods in that element-specific analysis of small samples can be achieved at close to *in situ* conditions (e.g. pH, temperature, moisture content and redox state), often at low concentrations in complicated environmental matrices (e.g. soils, precipitates and wastes). Both bulk and micrometre-scale XAS techniques (X-ray absorption near edge structure, XANES; extended X-ray absorption fine structure, EXAFS; μXAS; μXRD) and μXRF mapping are available at Diamond beamlines and constitute the main methods used by workers interested in understanding the environmental behaviour and fate of contaminants.

**Figure 1.**
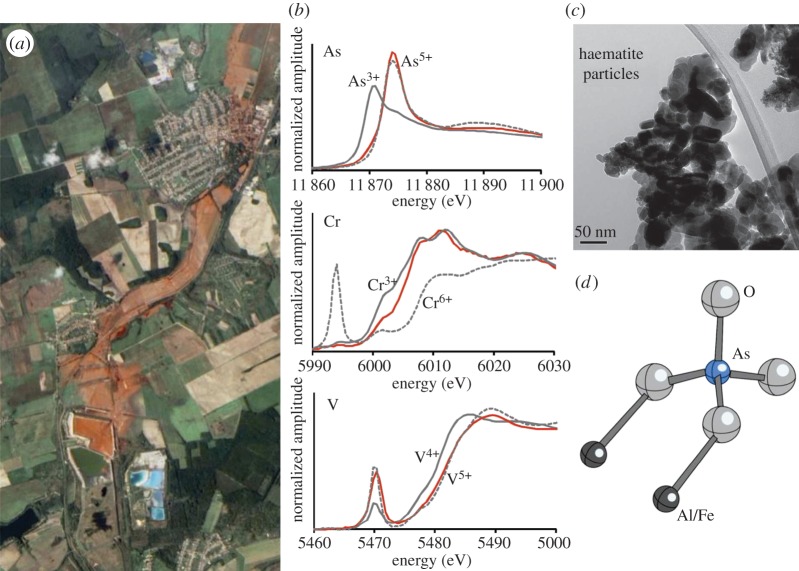
(*a*) The catastrophic failure of the bauxite residue dam at the Ajka alumina plant on 4 October 2010 resulted in the release of around 1 million m^3^ of caustic metalliferous red mud slurry (NASA Landsat image). In the weeks following the spill, there was much concern regarding the potential leaching of toxic contaminants, especially arsenic, chromium and vanadium, which are present at high concentrations in the red mud. XANES data collected at Diamond from beamline I18 (*b*) from samples of the red mud helped to characterize several of these potentially toxic elements, which was then used in conjunction with high-resolution (transmission electron microscopy) imaging (*c*) and molecule-scale modelling (*d*) of the phases present from EXAFS data to predict the long-term risks posed by the toxic trace elements present in the red mud. (Online version in colour.)

The bauxite residue (red mud) spill at Ajka, Hungary, was the first large-scale uncontrolled release of this material into a terrestrial environment; therefore, there was little information available on the likely long-term consequences of red mud addition to ecosystems [35–37]. Red mud produces highly alkaline leachate (up to pH 13 [38]) in which several oxyanionic-forming trace elements including Al (650 ppm), As, V and Mo (4–6 ppm) are very soluble [36,39] and red mud itself has elevated concentrations of As, V, Cr, Co, Cu and Ni (100–1000 ppm) [36]. XAS analysis at Diamond beamline I18 was used to investigate the speciation of As, Cr and V in samples recovered from the site (figure 1) [10]. Chromium in red mud was discovered to be effectively trapped in iron oxide nanoparticles and is very resistant to future remobilization. Arsenic and vanadium, however, exist as more easily solubilized arsenate and vanadate species. The potential to remove toxic elements (such as Al, As and V) from red mud leachates during neutralization reactions was also investigated [40], with XANES and EXAFS analysis used to characterize the fate of metal(loids) in precipitates formed during neutralization [41]. Although Al and As could be successfully removed from leachates by neutralization reactions, vanadate remained essentially soluble and high V concentrations are therefore likely to be an intractable problem during the treatment of red mud leachates. Current work has focused on the leaching of metal(liod)s from red mud affected soils [42], using XANES to characterize changes in As speciation under reducing conditions [43]. In addition to the study of bauxite residues, XAS analysis at Diamond has also been recently used to investigate the speciation of several different elements (As, P, Pb, Sb and Zn) in mine tailings and contaminated sediments, again helping to understand their environmental mobility and the associated risks to ecosystem health [44,45].

Another example from Diamond beamline I18 was the characterization of Cr speciation in soils found beneath a chromite ore processing residue (COPR) disposal site in the north of England. This soil contained a diverse population of anaerobic alkaliphiles and reduced Fe(II) [46,47], despite receiving a continuous influx of a Cr(VI) contaminated, hyperalkaline leachate (pH 12.2). Chromium was found to have accumulated in this soil as a mixed Cr(III)–Fe(III) oxyhydroxide phase [48] produced as a result of an abiotic reaction of Cr(VI) with Fe(II) present in the soil [49]. This soil-associated Fe(II) is therefore acting as a natural reactive zone beneath the COPR and thereby preventing the spread of Cr(VI) in the environment.

## 3. Environmental radioactivity and management of nuclear waste legacies

Management of the nuclear legacy within the UK and worldwide is one of the most important environmental issues of the coming decades. This includes the disposal of radioactive waste within geological disposal facilities, decommissioning of legacy nuclear sites and strategies for management of potential nuclear incidents. These projects require a considerable amount of fundamental research, including studies of radionuclide interactions with environmental materials (e.g. minerals, microorganisms and natural waters) to predict their mobility and speciation in the environment. Also, a detailed knowledge of containment and treatment strategies, which could be used to dispose of radioactive waste and clean up contaminated sites, is required. The delivery of this research requires a variety of approaches and techniques, including those which can characterize the distribution of radionuclides in complex heterogeneous environmental materials (e.g. soils) and determine radionuclide speciation (e.g. oxidation state) at the low mass concentrations at which radionuclides are found in environmental systems. The facilities at Diamond have enabled a step change in research capability and allowed new research directions which directly address the challenges associated with the long-term management of the UK's nuclear legacy.

**Figure 2.**
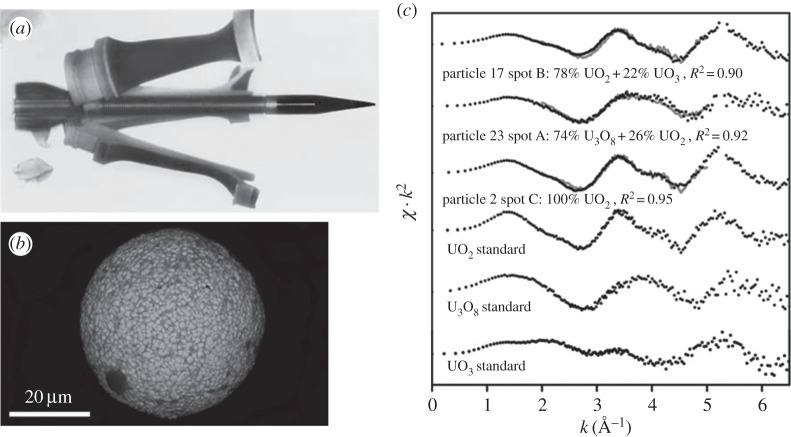
The use of depleted uranium (DU) in battlefiled munitions has led to much interest in the fate and behaviour of DU in the environment, especially with respect to its potential solubility and health effects. (*a*) DU penetrator rod used in battlefield munitions (adapted from [51]); (*b*) scanning electron microscopy image of uraniferous (uranium oxide) particle from dust/soil samples, showing primary morphology and (*c*) μEXAFS spectra for three U-oxide standards and three selected sample spectra from U-containing dust samples showing that the particles are a mixture of UO_2_ and UO_3_ (adapted from [50]).

Depleted uranium (DU) metal is used for a variety of purposes, including kinetic energy penetrators, tank armour and radiation shielding. Environmental contamination by DU has been identified in a number of sites globally as a legacy of DU metal component manufacturing, and use of DU munitions. Key to predicting the long-term behaviour of uranium at these sites is characterizing the chemical form (speciation) of the uranium in the soil/sediment. μXAS using beamline I18 at Diamond was used [50] to study samples collected adjacent to a U metal processing plant (New York, USA). Uranium-rich microspheres (20–80 μm) were analysed by a combination of μXANES and μEXAFS (figure 2). Linear combination fitting of μEXAFS data showed that the particles consisted of uranium oxide, dominated by UO_2_ with variable amounts of U_3_O_8_. This indicated that the uranium within the particle was mostly U(IV) which is less mobile and less bioavailable than the U(VI) species, which are highly soluble under oxic conditions. This work highlights the importance of microfocus techniques for studying radionuclides in natural samples where the overall concentration of the radionuclide is low, and contaminant behaviour is controlled by a small number of highly enriched particles.

The mobility of radionuclides in the subsurface is often controlled by adsorption and incorporation into environmental mineral phases, including iron oxides (e.g. haematite, Fe_2_O_3_) and carbonates (e.g. calcite, CaCO_3_), and/or by biologically mediated processes. A molecular-level understanding of the mechanism of mineral sorption and the changes in radionuclide speciation which occur during microbial processes are key to determining their fate and mobility. Work at Diamond [52], which builds on previous synchrotron studies [53–56], has used XAS to show that microbially mediated Fe(III) reduction in sediments is linked to the bioreduction of key radionuclides (e.g. U and Tc). This leads to the formation of highly insoluble U(IV) and Tc(IV) phases (e.g. UO_2_ and TcO_2_). These processes have the potential to be used in bioremediation to limit Tc and U mobility in contaminated land. However, it is important to note that even when reduced to U(IV), uranium can still be mobile. Wang *et al*. [21] used XAS to show that U(IV) is being transported via colloids in a mine impacted wet land (France). EXAFS analysis was used to support findings that U(IV) was bound to Al–P–Fe–Si aggregates, Fe and organic matter colloidal particles. The U(IV) was transported with these particles within soil, and ultimately released into surface water sources (i.e. rivers).

Many of the intermediate level waste geodisposal facilities currently being planned or constructed include the use of cement in waste forms, backfill and in the construction of the repository. Alkaline pore water from the cementious repository will react with the surrounding geosphere forming a chemically disturbed zone (CDZ). The UK NERC-funded BIogeochemical Gradients and RADionuclide transport (BIG-RAD; www.bigradnerc.com; 2011–15) project has used the facilities at Diamond to investigate the behaviour of radionuclides within the CDZ. EXAFS studies using B18 have shown that the speciation of U sorbed to the surface of silicate minerals in the CDZ changes significantly with pH (10.5–13) [57]. In addition, U has been shown to become incorporated within haematite when formed under CDZ conditions [58]. Finally, bioreduction of U(VI) to U(IV) has been reported [59] up to pH 10, indicating biologically mediated processes may be important within the CDZ. Overall, these studies have shown that the behaviour of radionuclides in the CDZ is complex, and controlled by a variety of geochemical, mineralogical and biological factors. This type of fundamental insight into radionuclide behaviour could be used to inform the safety case for future geodisposal facilities.

The research being undertaken at Diamond is world-leading in many areas and is complemented by research from synchrotron facilities outside the UK which specialize in the analysis of radioactive materials. These include the Rossendorf beamline at the European Synchrotron Radiation Facility (http://www.esrf.eu/UsersAndScience/Experiments/CRG/BM20) [60] and the Institute for Nuclear Waste Disposal beamline, Angströmquelle Karlsruhe (ANKA; http://www.anka.kit.edu/english/981.php) [61], which have advanced containment and control facilities that allow the analysis of highly active samples, including those containing transuranic elements (e.g. Pu). There are new beamlines at Diamond to come online (see table 1 for details) which will expand capability within environmental radioactivity research. The spectroscopic analysis of samples containing low levels of radionuclides (tens of ppm) is now possible on the new ultra dilute spectroscopy beamline (I20), which will enable speciation analysis at levels approaching those which may be present in a contaminated environment. In addition, new nanofocus facilities will enable fluorescence and XANES mapping at a 20–30 nm resolution (I14 and I08), which could allow the distribution and speciation of radionuclides associated with individual soil components (e.g. mineral grains, microorganisms and water within soil pores) to be determined. Finally, the development of environmental radiochemistry research at Diamond is now being supported by the UK Science and Technology Facilities Council funded Environmental Radioactivity Network (www.enradnet.co.uk; 2012–2015). This programme will seek to increase the range of radionuclides which can be analysed (e.g. transuranic elements) and developing the use of *in situ* techniques including grazing incidence XAS [62] and time-resolved SAXS and wide angle X-ray scattering (WAXS).

## 4. Understanding natural environmental processes (past and present)

Understanding natural environmental processes very often involves understanding and subsequently predicting the reactivity of trace elements in the environment. The cycling of these elements couples the lithosphere, hydrosphere, biosphere and atmosphere and is fundamental to contaminant toxicity and migration (see above), the abundance and distribution of elements that are essential for life and the formation of economically attractive ore deposits [11]. By definition, these species are typically found at very low abundance and are often heterogeneously distributed in environmental media. Understanding their biogeochemistry, however, is vitally important because many trace metals are bio-essential at natural concentrations but toxic to life in excess. Environmental geoscientists are therefore concerned with the factors that control their fate and mobility in the environment. In many cases, this involves detailed investigation of the biogeochemical interactions that occur between dissolved trace metals in surface and subsurface fluids and the solid components of soils, sediments and rocks, namely minerals and both abiotic and biotic organic matter. At these interfaces, dissolved trace metals can be concentrated via, often coupled, sorption and redox processes, which can lock up toxic contaminants in a relatively harmless chemical form, or similarly remove bio-essential elements from the bioavailable pool. To understand the biogeochemical processes that control the reactivity and cycling of trace elements, there is an arsenal of both traditional and cutting-edge tools available. The macroscopic interactions of trace metals with environmental media can often be elucidated with careful laboratory experiments which are typically coupled to various geochemical modelling approaches (e.g. [63–65]), but to determine the processes and mechanisms that ultimately control trace metal toxicity and bioavailability microscopic insight into environmental systems is increasingly required.

**Figure 3.**
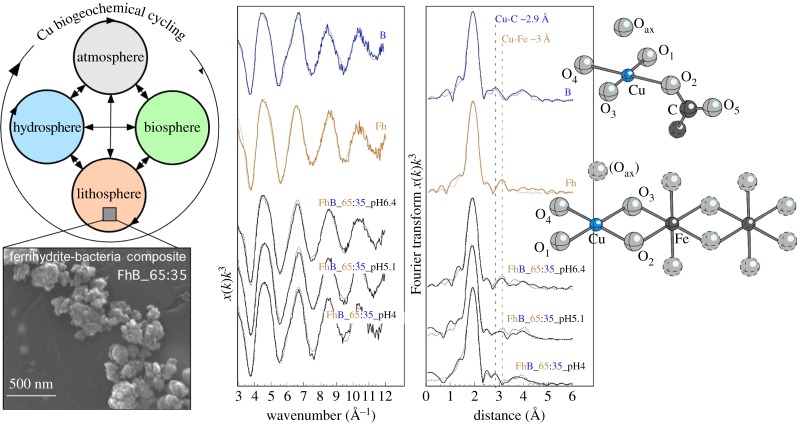
Iron oxide–bacteria composites (scanning electron microscopy image bottom left-hand side) are widespread in natural environments and are extremely efficient scavengers of bio-essential trace metals such as Cu. (Cu biogeochemical cycle shown top left-hand side.) EXAFS data (adapted from [20]; centre) were collected from Cu adsorbed to bacterial cells (*Bacillus subtilis*, B), hydrous ferrous oxide (ferrihydrite, Fh), and adsorbed to several iron oxide–bacteria composites at Diamond on beamline B18. These data were fit to molecular clusters (right-hand side) to show that Cu binding to carboxyl functional groups present on the bacterial cell wall plays an important role in adsorbing Cu and thus in controlling its environmental fate and mobility (where the bacterial fraction binds all of the adsorbed Cu at pH 4, and a significant proportion of the adsorbed Cu at pH 5–7). (Online version in colour.)

Environmental process research at Diamond has significantly extended our understanding of contaminant and bio-essential trace metal behaviour in the environment. These studies recognize the important role of both inorganic and organic sorbents, and the complex interplay between these soil and sediment constituents, and for this reason they are expanding the frontiers of environmental geoscience. In particular, microfocus X-ray diffraction (μXRD), fluorescence (μXRF) and absorption spectroscopy (μXAS) on beamline I18 were among the first to shed light on the important role of earthworms, and the calcium carbonate mineral granules excreted by these organisms, in regulating the fate and mobility of lead and strontium [66,67], and in the bio-accumulation and bioavailability of metals in earthworm tissues [68]. XAS on beamline B18 is also providing new insight into the role of mineral–organic composites in regulating the reactivity and cycling of bio-essential copper [69]. In this work, the iron oxide mineral ferrihydrite and ferrihydrite–bacteria composites were synthesized via rapid Fe(III) hydrolysis, with composite synthesis performed in the presence of the common soil bacteria *Bacillus subtilis*. Composites were synthesized with different ferrihydrite-to-bacteria mass ratios encompassing the range of ratios commonly found in natural environments. XAS reveals for the first time that copper uptake by these near-ubiquitous composites is the result of adsorption to both the ferrihydrite and bacterial fractions (figure 3). Furthermore, copper adsorbs to the composite fractions via the same molecular mechanisms as to the end-member ferrihydrite and bacterial phases, namely an inner-sphere bidentate edge-sharing surface complex on the ferrihydrite fraction [69] and an inner-sphere monodentate surface complex to carboxyl functional groups present on the cell walls of the bacterial fraction [70]. Overall, for composites dominated by either ferrihydrite or *B. subtilis*, the bacterial fraction is exclusively responsible for copper adsorption at low pH, whereas the ferrihydrite fraction is predominantly responsible for adsorption at high pH. When combined with previous work investigating the uptake of copper onto bacteria [70], humic substances [71,72] and iron (hydr)oxides coated with humics [73,74], this work reveals that the fate and mobility of copper in both natural and contaminated soils is controlled by its strong adsorption to carboxyl functional groups that are present in microbes, soil humic substances and mineral–organic composites.

Diamond research is also among the first to investigate traditionally macroscopic processes at the microscopic scale, including shale degradation and associated coastal erosion [75], the colonization of seafloor basalts [76] and the mechanical behaviour of frozen soil linked to subsurface fluid migration using tomography on beamline I12 [16]. At the interface of environmental and planetary research, Diamond users are also providing new insight into mineral phases important for star and planetary formation [77,78] and in volcanology for the prediction of super-eruptions [79].

In a relatively new application of synchrotron techniques, work at Diamond is also starting to shed light on the microscopic processes responsible for creating and preserving elemental signatures in the rock record. In this sense, Diamond users are among the first researchers to provide molecular explanations for elemental trends that are frequently observed in geological deposits. These signatures, including metal concentrations and stable isotope compositions, reflect the chemical composition of the fluids from which the original sediments were deposited, and so can be used to trace palaeo biogeochemical processes in freshwater and seawater. For example, μXRF and μXAS on beamline I18 reveal for the first time the coupled sorption and redox processes responsible for preserving thallium concentrations and stable isotope compositions in ancient ferromanganese-rich ocean sediments. Work shows a sorption–oxidation–fractionation molecular mechanism, whereby monovalent thallium in seawater adsorbs to manganese oxide, is oxidized to trivalent thallium at the manganese oxide surface, which induces an isotope fractionation between ^203^Tl and ^205^Tl, with the heavier isotope concentrating in the oxidized species [80]. In this mechanism, the proportion of the manganese oxide hexagonal birnessite dictates the extent of thallium oxidation, and thus the extent of thallium enrichment and isotope fractionation. Changes in thallium signatures preserved in ocean sediments over the last approximately 80 million years might reflect perturbations in the rate of oceanic organic carbon burial, and thus the rate of oceanic CO_2_ sequestration [81].

## 5. Characterizing mineral precipitation and weathering reactions

The making of bonds during the formation of mineral phases from solutions or melts or the transformation of minerals during various geological processes, as well as the breaking of bonds in minerals during rock weathering usually follow a series of complex steps and pathways that most often depend on molecular-level reactions occurring at mineral–fluid–microbe interfaces. In many cases, mineral formation reactions involve the initial precipitation of amorphous or poorly ordered nanoparticulate phases from solution. These then crystalize to more thermodynamically stable mineral phases and during this crystallization process they may sequester harmful elements. In the reverse reactions, during mineral weathering, the release of elements from minerals, regardless if in abiotic or biotic systems, governs a large part of the terrestrial and marine carbon cycles and also the rate of soil formation. The application and use of synchrotron radiation light to elucidate environmental mineral formation and breakdown processes has revolutionized our understanding of such reactions. The capabilities of Diamond beamlines have allowed the application of synchrotron-based scattering, diffraction and spectroscopic techniques to gain a detailed in sight into many environmentally relevant reactions.

**Figure 4.**
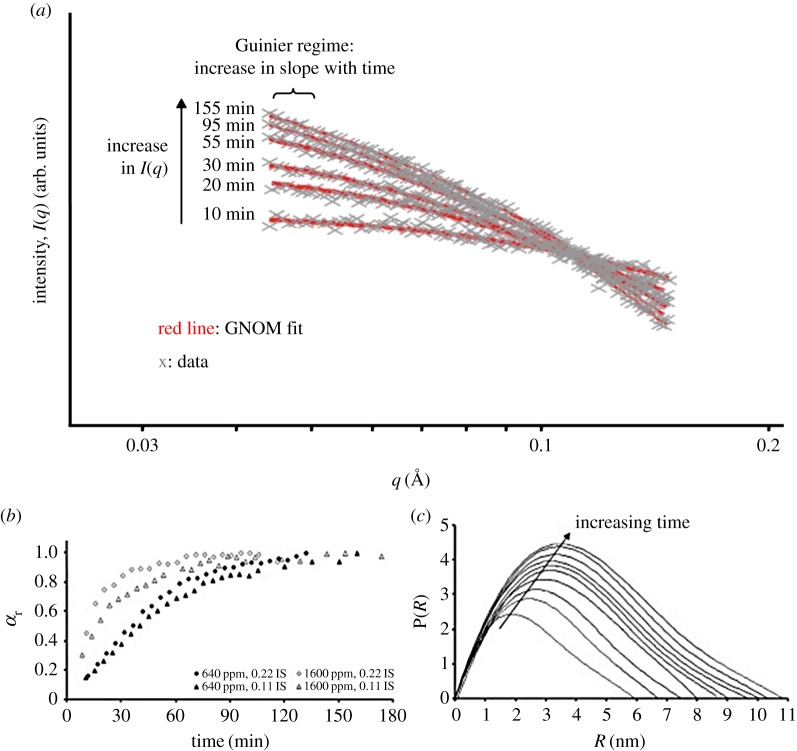
(*a*) The nucleation and growth of silica nanoparticles in solution as evidenced through the change in scattering intensity in an *in situ* and time-resolved SAXS experiment; (*b*) time evolution of normalized scattering intensity at various conditions; and (*c*) pair distribution function P(*R*) of scattered silica nanoparticles as a function of radius *R* (in nm) and time (adapted from [82]). (Online version in colour.)

The first step in any solution-based coalescence of ions to form a de novo phase is often simple hydrolysis or polymerization. The formation of amorphous silica proceeds through a polymerization of single monomeric SiO_2_ entities and this leads to the formation of a wide-open, highly hydrous nanostructure. Bespoke experimental systems have been designed to make use of synchrotron-based *in situ* and time-resolved SAXS capabilities to quantify the kinetics and mechanisms of amorphous silica formation [82,83] (figure 4). These studies aimed to mimic how silica precipitation occurs in natural systems (e.g. geothermal hot springs, diatoms or natural marine sediments). Once polymerization was induced a fast decrease in monomeric silica in solution was accompanied by a simultaneous increase in scattering intensity through the formation of 1–2 nm silica nanoparticles. With time these grew to reach a final size of 7–8 nm. The nucleation and growth of silica nanoparticles from supersaturated solutions follow a three-stage process that proceeds through the homogeneous and instantaneous nucleation of these initial nanoparticles followed by a three-dimensional surface-controlled particle growth following first-order reaction kinetics and in a latter step through Ostwald ripening and particle aggregation.

**Figure 5.**
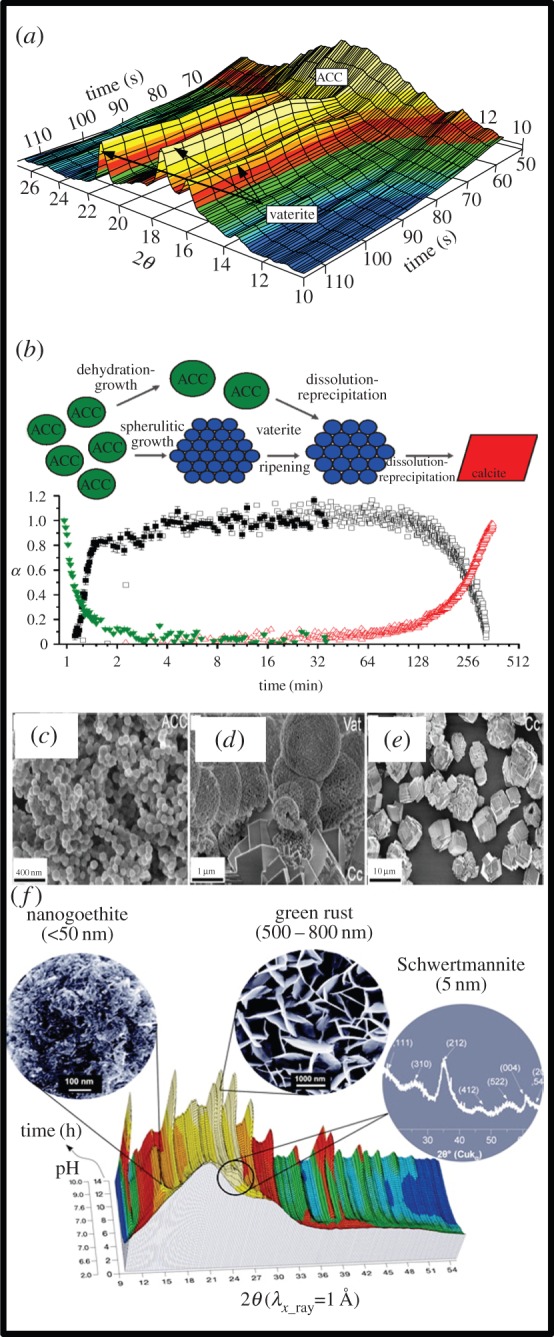
(*a*) Three-dimensional WAXS plot of the fast transformation of ACC to vaterite; note time axis at right. (*b*) Schematic of the multistage ACC → vaterite → calcite crystallization pathway (adapted from [84]). (*c*–*e*) Electron microscope microphotographs of solids quenched throughout a full ACC to calcite transformation reaction. (*f*) Three-dimensional plot and corresponding images and diffraction patterns as insets showing an *in situ* time-resolved WAXS experiment that followed the formation of GR through the various stages and intermediate phases (schwertmannite and nanogoethite). (Adapted from [12].) (Online version in colour.)

The formation of calcium carbonate phases by living organisms (e.g. coccoliths) in marine system is a key part of the global carbon cycle, which in turn is key to controlling the chemistry of our oceans (e.g. pH) and atmosphere (e.g. pCO_2_). Amorphous calcium carbonate (ACC) is a transient intermediate phase in the formation of many crystalline calcium carbonate minerals, in particular during biomineralization. ACC formation and crystallization have been studied using *in situ* and time-resolved SAXS/WAXS on station I22 at Diamond that were only possible due to the fast time resolution capabilities of this station (1 s per frame). The crystallization of pure ACC to vaterite (μ-CaCO_3_) occurs in supersaturated solutions in less than 90 s [84] (figure 5). The 35–40 nm ACC nanoparticles dehydrate and dissolve and concomitantly vaterite forms via a nucleation-controlled mechanism to form initially 9 nm particles (2 min) which grow to form micrometre-sized spherulites consisting of individual nanoparticles that are 50–60 nm in size (15 min). In a subsequent stage, these vaterite spherulites transform to calcite, via a slower (hours) dissolution and re-precipitation mechanism [85]. An alternative pathway that leads to the direct conversion of ACC to calcite with no vaterite intermediate can occur through doping of the initial ACC with Mg [86] (figure 5*f*). Recent studies [87,88] have extended this work (using XAS on beamline I18 and high-resolution XRD on beamline I11) to show that the presence of organic molecules is key to controlling the ACC crystallization pathway and to describing the high temperature/pressure transformation of ACC to vaterite in systems equivalent to extraterrestrial environments [89,90].

Iron minerals and their formation and inter-transformations in terrestrial, atmospheric or marine settings control many other element cycles. This is due to their often nanoparticulate nature and thus high surface area and reactivity but also due to the fact that they are most often involved in multiple redox reactions both in solution and solid phases. For example, iron sulfides (mackinawite, greigite and pyrite) play a fundamental role in sedimentary environments, ore deposits and heavy metal immobilization. However, their solution-based formation and transformation has only in the last decade been addressed using synchrotron-based *in situ* and time-resolved diffraction [91,92]. These studies have shown that following solution-based reactions in highly anoxic systems is feasible and that the crystallization of pre-formed, poorly ordered mackinawite (an Fe^2+^ only containing phase, nominal FeS) can, depending on redox conditions and additives, follow various pathways. Under highly reducing conditions, mackinawite often transforms first to a highly magnetic and Fe^2+^/Fe^3+^ bearing intermediate, greigite (Fe_3_S_4_) [92]. This ultimately recrystallizes and leads to the formation of the geologically stable pyrite (FeS_2_).

Iron oxide mineral phases play equally a crucial role in most environments and for example in sites contaminated with either inorganic (e.g. lead and chromium) or organic pollutants (e.g. trichloroethene), toxic compounds that are often difficult to remediate, they play a fundamental role in sequestering and cycling toxic compounds. Work at the former UK synchrotron site at Daresbury laboratory and continuing at Diamond has shown that the formation and inter-transformation of iron-bearing phases can control the fate of trace elements [12,93–95]. For example, the multistage reaction pathways during the solution-based formation of green rust (GR) were elucidated using the SAXS/WAXS capabilities of Diamond beamline I22. *In situ* time-resolved data collection was used with a specialized reactor system, in which the changing electrochemical solution characteristics (i.e. pH, Fe^2+^/Fe^3+^, redox, anaerobicity, etc.) could be precisely controlled and monitored (figure 5). GR phases form in suboxic conditions and are layered double-hydroxide phases that have the capacity to reduce a range of inorganic and organic species. At low pH (2.8–4.5), the first nanophase that forms directly from solution is schwertmannite (Fe_8_O_8_(OH)_4.5_(SO_4_)_1.75_). With increasing pH (greater than 5), adsorbed Fe^2+^ ions catalyse the transformation of schwertmannite to goethite (α-FeOOH). Finally, the hydrolysis of the adsorbed Fe^2+^ ions on goethite initiates its transformation to GR at pH > 7 [12].

**Figure 6.**
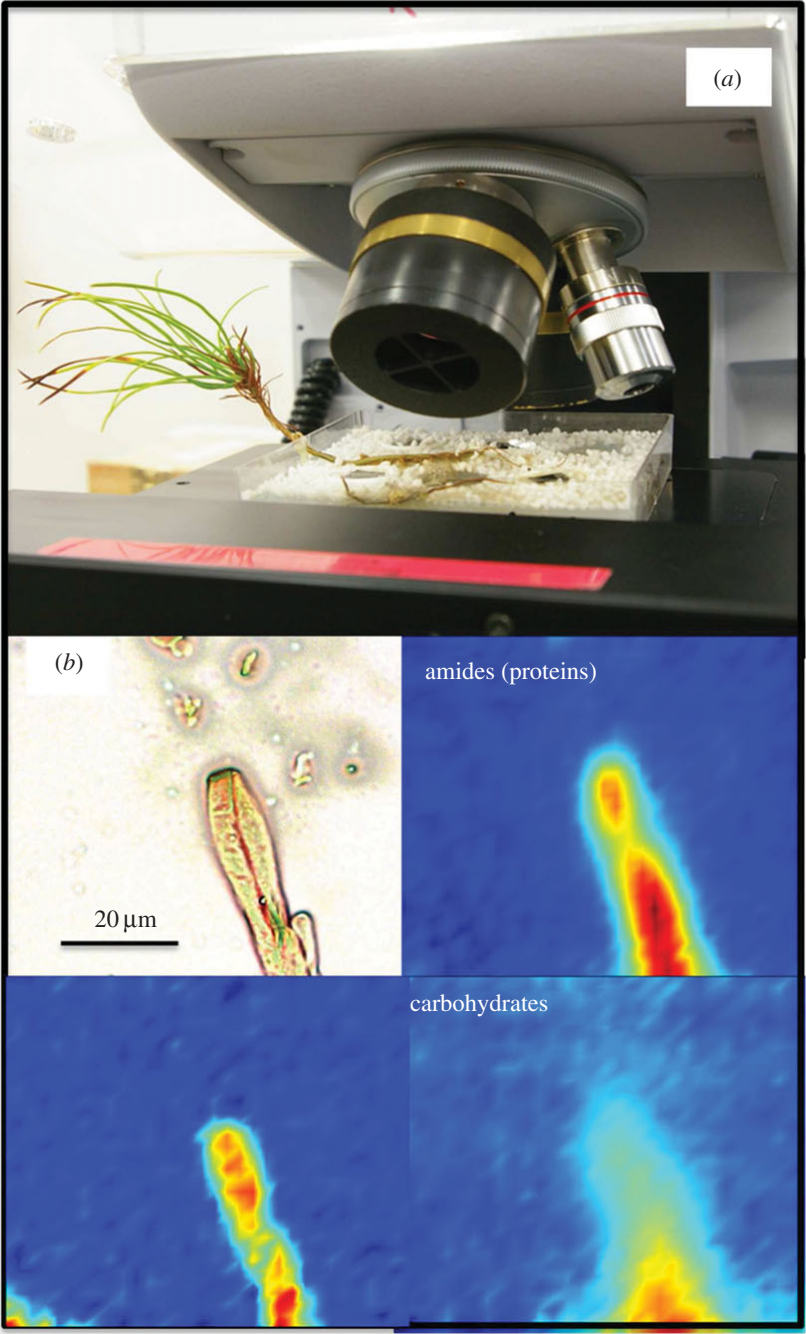
(*a*) A Scots pine (*Pinus sylvestris*) tree with its roots and symbiotic fungi (*Paxillus involutus*) in a microcosm as used for synchrotron-based μ-FTIR analysis at the Diamond Light Source beamline B22. (*b*) Microscopic image and corresponding functional group distributions for proteins, lipids and carbohydrates in a hypha grown on a biotite surface in the microcosm shown in (*a*). (Online version in colour.)

Soils form through biotic weathering of rocks. Understanding weathering reactions is therefore important as humans currently destroy more soil than natural weathering processes can produce, yet we do not understand how to manage this. Crucially, synchrotron radiation techniques (using time-resolved IR microspectroscopy) have been developed to follow weathering processes at mineral–microbe interfaces, ideally with the microbes alive and not stressed [96]. Using the IR microspectroscopy beamline B22 at Diamond (figure 6), the detailed mapping of functional groups of living fungi that have grown on a nutrient-rich mineral substrates (biotite, the sole source of K in the system) was performed to understand the role they play in mineral breakdown [18]. This built on earlier work where combining synchrotron-based soft X-ray microspectroscopic analyses of the fungal–biotite interface with focused ion beam milled sections analysed in detail with high-resolution transmission and spectroscopy revealed that the breakdown of biotite was speeded up dramatically by the presence of fungi [97]. The weathering process starts with a biomechanical forcing of the mineral–fungal interface due to the colonization of the mineral surface by fungi. The mineral lattice is therefore weakened and subsequently chemical alteration and element removal (e.g. K removal and changes in Fe oxidation) leads to a transformation of the initial biotite to a K-free vermiculite-rich nanostructure. This work has shown that ectomycorrhizal fungi that are living symbiotically with trees extract nutrients from minerals by breaking the bonds faster than occurs abiotically.

## 6. Manufacture of functional (nano)materials for environmental applications

Functional nanomaterials often have useful physical attributes that differ from bulk properties, such as enhanced magnetic, electrical or chemical behaviours. There are chemical methods to produce nanoparticles; however these often use harsh chemicals, or extremes in physical treatment. Processes present in the environment have more recently been investigated as ‘green chemistry’ routes for the synthesis of a variety of different nanomaterials. Synchrotron radiation science is critical in the development of this important scientific area as it is crucial to identify and characterize the physical and chemical attributes of nanoparticles produced by these processes in order to better understand their potential in future commercial processes.

**Figure 7.**
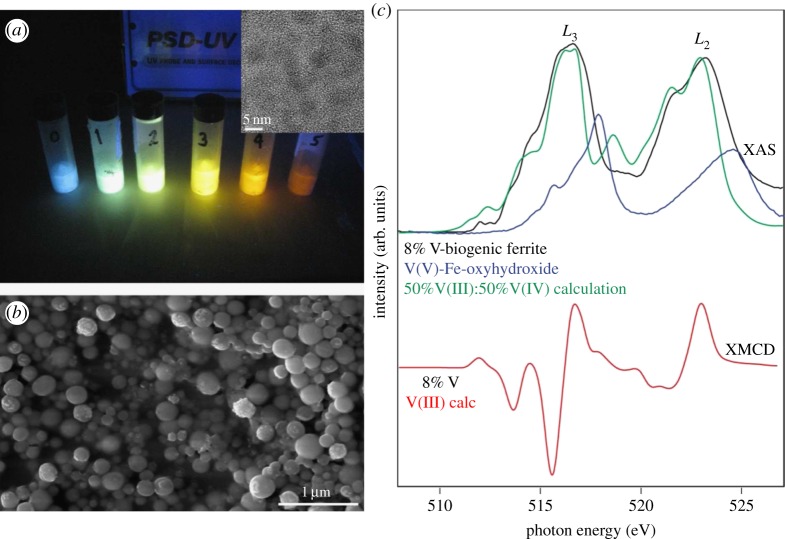
Environmental processes can be harnessed to create novel functional nanomaterials. For example, the bacterium *V. atypica* is able to produce (*a*) biogenic cadmium selenide (CdSe) quantum dots with changing particle sizes showing differing optical properties under UV light (inset transmission electron microscopy image of CdSe quantum dots) (adapted from [102]) and (*b*) environmental scanning electron microscopy image of biogenic selenium nanospheres also created by *V. atypica* and exposed to Hg showing a potential remediation strategy (adapted from [103]). Fe(III)-reducing bacteria, such as *Geobacter sufurreducens*, are able to create nanoparticles of magnetite (Fe_3_O_4_) and substitute different transition metals such as vanadium altering the physical properties of the nanoparticle as shown in the data taken at Diamond beamline I06 (*c*) at the V L2,3-edge, where both the XAS and XMCD of biogenic V–ferrite indicate that V(III) was incorporated into the biogenic spinel by the bacterial reduction of Fe(III) and V(V) creating novel nanoparticles (adapted from [104]).

Anaerobic bacterial reduction of metals and metalloids coupled to the oxidation of organic matter or hydrogen is well documented in the literature [98,99] and these powerful redox reactions can be channelled to produce useful materials. The bacterium *Veillonella atypica* can reduce aqueous selenate (Se^4+^) to selenide (Se^2+^), and the resulting selenide then used to form chalcogenide quantum dots such as ZnSe with optical and semiconducting properties [100,101]. Fellowes *et al*. [102] created a suite of CdSe quantum dots (2–4 nm) using selenide produced by *V. atypica* and stabilized the particle surface using glutathione (figure 7*a*,*b*). XAS analyses performed on I18 at Diamond suggested that the Se was structurally incorporated into the CdSe. These materials were shown to have increased stability compared with synthetic analogues. *Veillonella atypica* can also form nanospheres of selenium [105]. A novel application of these active nanoparticles was the ability of selenium to sequester volatile mercury [103]. Within museum collections mercuric chloride was previously used to preserve samples as it is an effective pesticide; however, over time the mercury evolves to Hg^0^ vapour; a health risk to staff. XANES data collected on B18 at Diamond were used to identify that remaining mercury in specimens was present as a mixture of HgCl_2_, cubic HgS and HgO. Bacterially produced selenium nanoparticles were shown to be efficient absorbents of the toxic mercury vapour and XANES indicated that the Hg was captured as HgSe [103].

Dissimilatory Fe(III)-reducing bacteria have previously been shown to form ferrite spinels where the product of ferrihydrite reduction is manipulated to contain additional transition metals, such as Co, Ni or rare earth elements, altering the magnetic properties of the nanomineral [106–108], for uses in technological and medical applications [109,110]. A technique crucial to understanding the structure of metal-substituted magnetites is X-ray magnetic circular dichroism (XMCD), available at beamlines I10 and I06 at Diamond, as it is able to determine the structure of the three different Fe environments in the ferrite spinel [111,112]. It is possible to substitute vanadium into the structure of magnetite using Fe(III)-reducing bacteria [104,113]. Fe and V *L*_2,3_-edge XAS measured at I06 (figure 7*c*) indicated that when bacteria reduced V(V)Fe(III) hydroxide, they formed a material that had both V^3+^ and V^4+^ associated with it, but V *L*_2,3_-edge XMCD indicated that only the V^3+^ was incorporated into the spinel. In addition, the Fe *L*_2,3_-edge showed that the V was substituting predominantly for Fe^3+^ in octahedral coordination. This showed a method of capturing V within the structure of the spinel ferrite.

Diamond has a new high-field magnet beamline on I10, which can be used to perform XMCD experiments. This magnet is able to reach magnetic fields of up to 14 T and samples can be cooled to 3 K, which will allow the study of environmental samples with weaker magnetic signals. In addition, a new beamline, the scanning X-ray microscope (SXM) on I08, will open up a whole new scientific area to the UK community. The SXM beamline will be able to image samples, providing elemental, species and oxidation state information using XAS, on a sub-micrometre scale (down to 20 nm). The benefit of SXM to the environmental community is that, as well as reaching absorption energies corresponding to important metals and metalloids such as Fe, Mn, Co, U and As, this beamline will also be able to image biological material at the C, P and N edges, concurrently. SXM has previously been used to image environmental magnetic nanoparticles and can be used to collect XMCD on individual nanoparticles, or clusters of particles [90,91]. Using a sample of nano-magnetite produced by an Fe^3+^-reducing bacterium, Coker *et al*. [113] were able to image the position of bacteria in relation to the magnetic nanoparticles and discern that the distance from the bacterium changed the ratio of Fe^2+^ : Fe^3+^ in the nano-magnetite.

## 7. Summary and future perspectives

From field observations and measurements much can be learnt about the complexity of environmental reactions occurring in natural settings. However, it is only by experimental confirmation and cross-validation of the specific speciation, reaction mechanisms and kinetics that the molecular-level processes that drive the biogeochemical cycles on the Earth and elsewhere can be fully elucidated. Diamond has provided Earth and environmental scientists, in the UK and elsewhere, with access to a cutting-edge, world-leading scientific facility to do this research. It is now possible to use complementary spectroscopy, magnetic, imaging, scattering and diffraction beamlines at Diamond to investigate the fundamental properties of natural materials more rapidly, accurately and with higher resolution than ever before. The wide range of research described above is a testament to the achievements of Diamond in environmental science during a period of very rapid instrument development and installation. The high quality of beamline scientists and support staff at Diamond must also be recognized, for without their skills and continual support, our work simply could not happen. In particular, it should be highlighted how users are supported from their initial idea for an experiment right through the application processes to sample preparation, data collection and interpretation.

There is yet more to come. New beamlines coming online at Diamond now or in the near future (table 1) will, for example, allow XAS measurements to be collected from samples containing order of magnitude lower concentrations of the element of interest, expanding the range of experiments and natural samples that can be analysed. New capabilities in SXM and the hard X-ray nanoprobe will increase both the length-scale resolution and range of elements (including many organic molecules) that can be imaged and analysed successfully. The new laboratory facilities for using radioactive samples at Diamond beamline are also important for improving UK capabilities in nuclear science. Diamond has collaborated successfully with user groups in the establishment of the already impressive range of beamlines. It has therefore also successfully built a community of Earth and environmental scientists who can be consulted on future scientific decisions and collaborated with on the continual improvement of its facilities. Therefore, the best is yet to come and research done at Diamond will be at the forefront of meeting the environmental challenges that face society in the decades to come.
